# Investigation of aminoglycoside resistance inducing conditions and a putative AmrAB-OprM efflux system in *Burkholderia vietnamiensis*

**DOI:** 10.1186/1476-0711-13-2

**Published:** 2014-01-06

**Authors:** Agatha N Jassem, Connor M Forbes, David P Speert

**Affiliations:** 1 Department of Pathology and Laboratory Medicine, University of British Columbia, 950 W 28th Avenue, Vancouver, British Columbia V5Z 4H4, Canada; 2 Department of Pediatrics, University of British Columbia, 950 W 28th Avenue, Vancouver, British Columbia V5Z 4H4, Canada; 3 Centre for Understanding and Preventing Infection in Children, Child & Family Research Institute, University of British Columbia, 950 W 28th Avenue, Vancouver, British Columbia V5Z 4H4, Canada

**Keywords:** *Burkholderia vietnamiensis*, Aminoglycoside, Azithromycin, Hydrogen peroxide, Efflux, AmrB, AmrR

## Abstract

**Background:**

*Burkholderia cepacia* complex (BCC) bacteria are highly virulent, typically multidrug-resistant, opportunistic pathogens in cystic fibrosis (CF) patients and other immunocompromised individuals. *B. vietnamiensis* is more often susceptible to aminoglycosides than other BCC species, and strains acquire aminoglycoside resistance during chronic CF infection and under tobramycin and azithromycin exposure *in vitro*, apparently from gain of antimicrobial efflux as determined through pump inhibition. The aims of the present study were to determine if oxidative stress could also induce aminoglycoside resistance and provide further observations in support of a role for antimicrobial efflux in aminoglycoside resistance in *B. vietnamiensis*.

**Findings:**

Here we identified hydrogen peroxide as an additional aminoglycoside resistance inducing agent in *B. vietnamiensis*. After antibiotic and hydrogen peroxide exposure, isolates accumulated significantly less [^3^H] gentamicin than the susceptible isolate from which they were derived. Strains that acquired aminoglycoside resistance during infection and after exposure to tobramycin or azithromycin overexpressed a putative resistance-nodulation-division (RND) transporter gene, *amrB*. Missense mutations in the repressor of *amrB*, *amrR*, were identified in isolates that acquired resistance during infection, and not in those generated *in vitro*.

**Conclusions:**

These data identify oxidative stress as an inducer of aminoglycoside resistance in *B. vietnamiensis* and further suggest that active efflux via a RND efflux system impairs aminoglycoside accumulation in clinical *B. vietnamiensis* strains that have acquired aminoglycoside resistance, and in those exposed to tobramycin and azithromycin, but not hydrogen peroxide, *in vitro*. Furthermore, the repressor AmrR is likely just one regulator of the putative AmrAB-OprM efflux system in *B. vietnamiensis.*

## Findings

Members of the *Burkholderia cepacia* complex (BCC) can cause severe respiratory infections in individuals with cystic fibrosis (CF) [1]. Furthermore, many strains are highly and intrinsically resistant to various antimicrobials, including aminoglycosides [2], ribosome-targeting antibiotics important in the treatment of CF respiratory disease [3].

*B. cenocepacia* studies suggest that resistance-nodulation-division (RND) efflux systems are involved in BCC resistance to aminoglycosides [4-6]. The MexXY-OprM RND pump is the predominant determinant of aminoglycoside resistance in CF isolates of *Pseudomonas aeruginosa*[7], and aminoglycoside susceptibility in *B. pseudomallei* results from loss of AmrAB-OprA [8]. At subinhibitory concentrations, ribosome-targeting antibiotics and oxidative stress induce *mexXY* expression [9,10]. *mexXY* is under the control of the MexZ repressor [11], and *mexZ* mutations are common in pan-aminoglycoside resistant isolates [12].

We previously reported that *B. vietnamiensis* isolates are often aminoglycoside-susceptible and strains acquire resistance during chronic CF infection and under tobramycin and azithromycin pressure *in vitro*[13]. Decreased access of aminoglycosides to their target resulted from apparent gain of antimicrobial efflux via a RND pump, the latter determined with an inhibitor [13].

### *B. vietnamiensis* develops aminoglycoside resistance under hydrogen peroxide pressure *in vitro*

Aminoglycoside resistance can be induced in susceptible CF isolates of *B. vietnamiensis* following serial exposure to tobramycin (Table 1: C8395TE, D0072TE) or a single exposure to subinhibitory concentrations of azithromycin [13]. To characterize resistance inducing antimicrobial pressures further, after serial passage in cation-adjusted Mueller-Hinton broth (CAMHB) containing azithromycin, meropenem, ceftazidime, and co-trimoxazole at doubling concentrations as described previously [13] the drug susceptibility of C8395 was evaluated. Triplicate minimum inhibitory concentrations (MICs) were determined using broth microdilution methods [14], and their stability confirmed after 20 passages on antibiotic-free media. *P. aeruginosa* and non-*Enterobacteriaceae* breakpoints were used in the absence of *B. cepacia* breakpoints. Only serial exposure of C8395 to azithromycin resulted in notable (≥4 fold) increases in aminoglycoside MICs (Table 1). By previously described methods [9], but with selective agar containing tobramycin at 2.5 times the MIC, serial exposure of C8395 to hydrogen peroxide at half the MIC resulted in a 16-fold stable increase in aminoglycoside MIC for C8395PE (Table 1). Other acquired resistance was also observed: after passage with all antimicrobials the MICs of the respective agents against C8395 increased greatly (Table 1: C8395AE, C8395ME, C8395CE, and C8395SE), and some other cross-resistance, most notably between the β-lactams antibiotics, was also seen.

**Table 1:** **Antimicrobial susceptibilities of *B. vietnamiensis *** **after serial exposure to antibiotics or hydrogen peroxide**

**Isolate**^***a***^	**MIC (μg/ml)**^***b***^								
	**AMK**	**GEN**	**KAN**	**TOB**	**AZM**	**MEM**	**CAZ**	**SXT**	**CIP**
Clinical CF									
C8395 (3/11/1998, Bv1)	2	4	2	2	32	1	4	2/10	1
D0774 (25/7/2003, Bv1)	>128	128	128	128	>2048	128	128	64/320	>32
D0072 (15/03/2002, Bv3)	2	4	1	2	32	0.5	2	2/10	1
D2910 (31/03/2008, Bv3)	128	32	64	32	>32	2	4	1/5	16
*In vitro* exposed									
C8395TE (TOB)	>128	>128	128	>128	64	1	4	4/20	4
C8395AE (AZM)	32	16	16	16	2048	2	16	8/40	4
C8395ME (MEM)	16	8	8	8	32	16	64	4/20	16
C8395CE (CAZ)	8	8	4	4	32	8	16	2/10	16
C8395SE (SXT)	8	8	2	2	32	0.5	4	>64/320	8
C8395PE (peroxide)	32	64	32	32	32	4	16	4/20	4
C8395PC (control)	8	8	4	8	32	1	4	1/5	1
D0072TE (TOB)	32	32	16	16	>32	1	2	2/10	1

^a^Patient identification numbers and bacterial isolation dates are noted in brackets. *Abbreviations*: TE, TOB exposed; AE, AZM exposed; ME, MEM exposed; CE, CAZ exposed; SE, SXT exposed; PE, hydrogen peroxide exposed; PC, passage control.

^b^Aminoglycoside and azithromycin MICs for C8395 and D0774, and tobramycin and azithromycin MICs for D0072, D2910, C8395TE, and D0072TE were previously published to some extent [13] and are shown here for comparison. MICs represent susceptibility after 3 passages on antibiotic-free media. Abbreviations: AMK, amikacin; GEN, gentamicin; KAN, kanamycin; TOB, tobramycin; AZM, azithromycin; MEM, meropenem; CAZ, ceftazidime; SXT, co-trimoxazole; CIP, ciprofloxacin.

Hydrogen peroxide is, therefore, an additional inducer of aminoglycoside resistance in *B. vietnamiensis in vitro*, a particularly important finding because CF airways are rich in reactive oxygen species [15]. Moreover, *B. vietnamiensis* can acquire resistance after exposure to other antimicrobials used in treating BCC-infected CF patients, namely meropenem, ceftazidime, and co-trimoxazole [16].

The aminoglycoside-resistant derived isolates C8395TE and C8395PE accumulated 2.65 and 3.50 times less [^3^H] gentamicin than C8395, respectively (*P* = 0.0118, one-way ANOVA) (data not shown). Accumulation was determined in triplicate in Luria-Bertani (LB) medium as previously used to show the late, aminoglycoside-resistant isolate D0774 accumulates less gentamicin than C8395 [13]. There were no significant differences in the CFU/ml between C8395 and the comparison isolates at starting time (data not shown). Decreased access of aminoglycosides to their intracellular target is, therefore, responsible for the observed *in vitro* antibiotic and oxidative stress-induced resistance.

### Analysis of putative efflux system genes in clinical and *in vitro* stress exposed *B. vietnamiensis* isolates

Of the 11 putative RND transporters that the sequenced environmental *B. vietnamiensis* isolate G4 (accession NC_009256.1) contains (determined as previously [17]), following sequence alignment only Bcep1808_1575 showed high identity, 71%, 85%, and 92%, with the characterized transporters MexY (accession NC_008463.1) and AmrB (accession NC_007434.1), and their homologue BCAL1675 in *B. cenocepacia* (accession NC_011000.1), respectively. These transporters are part of an operon also encoding a repressor, membrane fusion protein, and outer membrane channel [7]. PCR product analysis revealed that *B. vietnamiensis* clinical isolates C8395, D0774, D0072, and D2910 contained these efflux system genes in the same order (data not shown).

To evaluate the expression of RND pump genes in *B. vietnamiensis*, triplicate overnight cultures were diluted 1:100 into CAMHB, LB medium, or synthetic cystic fibrosis sputum medium (SCFM) [18] with or without tobramycin and ceftazidime at half MIC or azithromycin, meropenem, and co-trimoxazole at a quarter MIC, and grown to an optical density at 600 nm (OD_600_) of 0.5 or 0.8. RNA was extracted using an RNeasy Plus Mini kit (Qiagen, Toronto, Canada), and treated with RNase-free DNase (Promega, San Luis Obispo, USA). Reverse transcription was performed using SuperScript II Reverse Transcriptase (Invitrogen, Carlsbad, USA) according to the manufacturer’s protocol. Amplification of the resultant DNA was quantified in a 7300 Real-Time PCR System (Applied Biosystems, Carlsbad, USA) in the presence of SybrGreen (Invitrogen), with primers for *Bcep1808_1575* (5’-CCGAACGACATCTACTTCAAGGTCGG-3’, 5’-ATCCTTCGCGACTTCGACGATCAG-3’), *Bcep1808_1573* (putative repressor gene) (5’-TGCAGATCCTGCGGATCACGAAA-3’, 5’-TTCGAGCAACGACACCAGATAGACG-3’), and *16S* (for normalization) (5’-CACGCTTTACGCCCAGTAATTCCG-3’, 5’-CCGGAAGAATAAGCACCGGCTAAC-3’). Denaturation occurred at 95°C for 10 minutes, followed by 40–50 cycles of 15 seconds at 95°C and 1 minute at 60°C.

**Figure 1:**
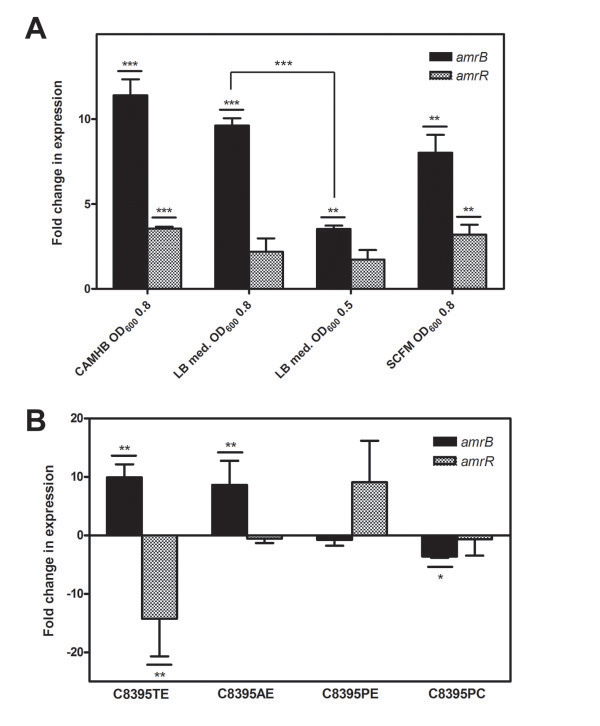
**Expression of the putative RND efflux system genes *amrB *and *amrR *in clinical CF and *in vitro *antibiotic or hydrogen peroxide exposed *B. vietnamiensis *isolates.** Expression was determined by real-time reverse transcription PCR and compared **(A)** between the early, aminoglycoside-susceptible isolate C8395, and the late, aminoglycoside-resistant D0774, in various types of media and stages of growth, and **(B)** between C8395 before and after its exposure to various antimicrobials, peroxide, or passage alone in CAMHB. The averages of three technical repeats were taken for each biological replicate. Fold change means were calculated by comparing the mean expression in C8395 to each biological replicate of **(A)** D0774 or **(B)** condition. Data points represent the averages of three biological replicates ± standard errors. **(A)** **, *P* < 0.01; *** *P* < 0.001 by unpaired Student’s t-test. **(B)** *, *P* < 0.05; **, *P* < 0.01 by Dunnett’s Multiple Comparison Test after one-way ANOVA. Abbreviations: *amrB, Bcep1808_1575*; *amrR*, *Bcep1808_1573*; RND, resistance-nodulation-division; CAMHB, cation-adjusted Mueller-Hinton broth; LB, Luria-Bertani medium; SCFM, synthetic cystic fibrosis sputum medium; OD_600_, optical density at 600 nm.

The late, aminoglycoside-resistant isolate D0774 expressed 11.4-, 9.6-, and 8.0-fold more *Bcep1808_1575*, herein “*amrB*”, than the early, aminoglycoside-susceptible isolate C8395, at OD_600_ of 0.8 in CAMHB, LB medium, and SCFM, respectively (*P* < 0.01) (Figure 1A). *amrB* expression between D0774 and C8395 was, however, 2.7 times less at OD_600_ of 0.5 vs 0.8 (*P* < 0.001) (Figure 1A), owing to D0774 expressing less *amrB* earlier (*P* < 0.01) (data not shown). Compared with C8395, D0774 also expressed 3.6- and 3.2-fold more *Bcep1808_1573*, herein “*amrR*”, at OD_600_ of 0.8 in CAMHB and SCFM, respectively (*P* < 0.01) (Figure 1A). In another set of sequential isolates, the late, aminoglycoside-resistant isolate D2910 also overexpressed *amrB* (by 5.3-fold) and *amrR* (by 2.4-fold) compared with the early, aminoglycoside-susceptible D0072 at OD_600_ of 0.8 in CAMHB (data not shown).

After a single exposure of C8395 to subinhibitory concentrations of test antimicrobials in CAMHB, *amrB* expression did not change (data not shown). After exposure of C8395 to serially doubling concentrations of tobramycin or azithromycin but not to other antibiotics or hydrogen peroxide (i.e. in C8395TE and C8395AE), expression of *amrB* increased 9.9- and 8.6-fold, respectively (*P* < 0.01) (Figure 1B, data not shown). Compared with C8395, C8395TE also expressed 14.2-fold less *amrR* (P < 0.01) (Figure 1B).

Active efflux via a RND efflux system is, therefore, probably involved in the decreased drug accumulation observed in *B. vietnamiensis* strains that acquired aminoglycoside resistance during infection and after exposure to tobramycin and azithromycin *in vitro*, owing to the correlation between resistance and *amrB* expression. Other aminoglycoside resistance determinants exist, since exposure to hydrogen peroxide did not induce *amrB* expression, and future tests with minimal medium would demonstrate that absoluteness of these observations. As proposed previously [19], *amrB* upregulation only in response to ribosome-targeting agents suggests it is a response to this interaction, not to antibiotics *per se*. Moreover, *amrB* overexpression was not sufficient to cause resistance to non-aminoglycoside antibiotics, supporting the notion that they are not substrates for the putative AmrAB-OprA efflux system [13]. Lastly, there was no association between *amrR* expression and aminoglycoside resistance or *amrB* expression, as is also true for *mexZ*[20].

To determine if mutations in *amrR* were responsible for the observed overexpression of *amrB* in *B. vietnamiensis*, sequences of the putative repressor were examined. DNA isolation, PCR using Phusion High-Fidelity DNA Polymerase (New England Biolabs, Ipswich, USA) with specific primers (5’-TTCAAAGAGGTGTGGGCAGGA-3’, 5’-CCGAAACCCGTGTTGTTCATC-3’), and product analysis by agarose gel electrophoresis were done using standard protocols [21]. PCR products were purified with a Wizard SV Gel and PCR Clean-Up System (Promega) and cloned into One Shot TOP10 *E. coli* cells with a Zero Blunt TOPO PCR Cloning Kit (Invitrogen). Plasmid DNA was isolated using a QIAprep Miniprep Kit (Qiagen), and M13 primers amplified *amrR*. Resultant products were sequenced at the UBC Centre for Molecular Medicine and Therapeutics.

C8395 and D0774 *amrR* differed from that of G4 by two silent mutations (data not shown). The late, aminoglycoside-resistant isolate D0774 also contained a substitution at position 425 (T → C), that at residue 142 of the protein, in the suggested ligand binding alpha helix region [22], replaces a leucine with a proline. D0072 and D2910 *amrR* sequences also differed: at position 156, or amino acid residue 52 amid the predicted DNA and C-terminal ligand binding domains [22], there was a ~2000 bp insertion in the late, aminoglycoside-resistant D2910. Only silent mutations were observed in *amrR* among C8395, C8395TE, C8395AE, C8395PE, and C8395PC (data not shown).

The *amrR* mutations identified likely influenced the expression of the putative *B. vietnamiensis amrB* transporter gene. The change in D0774 AmrR may indirectly affect DNA binding to the transcription factor [22], while the large insertion within D2910 *amrR* likely inactivates the repressor altogether. As per the *in vitro* derived isolate findings, aminoglycoside-resistant *P. aeruginosa* isolates overexpressing *mexXY* without mutations in *mexZ* also exist [23,24].

In conclusion, in *B. vietnamiensis*, oxidative stress can induce aminoglycoside resistance, while active efflux via the putative AmrAB-OprM efflux system is likely involved in clinical and *in vitro* antimicrobial-induced aminoglycoside resistance. Such elucidation of resistance inducing conditions and resistance factors may improve therapeutic regimens against infection with this species. Additional mechanisms of aminoglycoside resistance should be investigated next. The contribution of resistance determinants to aminoglycoside inefficacy may explain the observed varied degrees of resistance.

## Availability of supporting data

The data supporting the results of this study is included within the article.

## Abbreviations

BCC: *Burkholderia cepacia* complex; CF: Cystic fibrosis; RND: Resistance-nodulation-division; CAMHB: Cation-adjusted Mueller-Hinton broth; MIC: Minimum inhibitory concentration; LB: Luria-Bertani; SCFM: Synthetic cystic fibrosis sputum medium; OD600: Optical density at 600 nm.

## Competing interests

The authors declare that they have no competing interests.

## Authors’ contributions

ANJ designed the study, performed and analyzed experiments not done by CMF, and wrote the manuscript. CMF serially exposed C8395 to hydrogen peroxide and performed most of the susceptibility and expression tests. DPS participated in the design and coordination of the study and critically reviewed the manuscript. All authors read and approved the final manuscript.

## Authors’ information

Part of this work was presented at the American Association for the Advancement of Science Annual Meeting, Vancouver, BC, Canada, 16 to 20 February 2012.
